# Excessive Sedation as a Risk Factor for Delirium: A Comparison between Two Cohorts of ARDS Critically Ill Patients with and without COVID-19

**DOI:** 10.3390/life12122031

**Published:** 2022-12-05

**Authors:** Frank Anthony Rasulo, Rafael Badenes, Yaroslava Longhitano, Fabrizio Racca, Christian Zanza, Mattia Marchesi, Simone Piva, Silvia Beretta, Gian Piero Nocivelli, Basil Matta, Daniel Cunningham, Sergio Cattaneo, Gabriele Savioli, Francesco Franceschi, Chiara Robba, Nicola Latronico

**Affiliations:** 1 Department of Anesthesiology, Intensive Care and Emergency Medicine, Spedali Civili University Hospital, 25121 Brescia, Italy; 2 Department of Medical and Surgical Specialties, Radiological Sciences and Public Health, University of Brescia, 25121 Brescia, Italy; 3 Department of Anesthesiology and Surgical-Trauma Intensive Care, Hospital Clínic Universitari, School of Medicine, University of Valencia, 46010 Valencia, Spain; 4 Department of Anesthesia and Critical Care Medicine—AON St. Antonio and Biagio and Cesare Arrigo Hospital, 15121 Alessandria, Italy; 5 Foundation of “Ospedale Alba-Bra Onlus”—Department of Emergency Medicine, Anesthesia and Critical Care Medicine, Michele and Pietro Ferrero Hospital, 12060 Verduno, Italy; 6 Department of Emergency Medicine, Policlinico Gemelli/IRCCS—University of Catholic of Sacred Heart, 30149 Rome, Italy; 7 Department of Anaesthesia and Intensive Care, Addenbrookes NHS Hospital, Cambridge University, Cambridge CB39DF, UK; 8 Division of Cardio-Thoracic Intensive Care, Spedali Civili Hospital, 25121 Brescia, Italy; 9 Emergency Medicine and Surgery, IRCCS Fondazione Policlinico San Matteo, 27100 Pavia, Italy; 10 PhD Program in Experimental Medicine, Department of Clinical-Surgical, Diagnostic and Pediatric Sciences, University of Pavia, 27100 Pavia, Italy; 11 Policlinico San Martino, Department of Surgical Sciences and Diagnostic Integrated, University of Genoa, 16100 Genoa, Italy

**Keywords:** SARS-CoV2, delirium, deep sedation, adult respiratory distress syndrome, electroencephalography

## Abstract

Excessive sedation is associated with poor outcome in critically ill acute respiratory distress syndrome (ARDS) patients. Whether this prognostic effect varies among ARDS patients with and without COVID-19 has yet to be determined. We compared the prognostic value of excessive sedation—in terms of delirium, length of stay in intensive care unit (ICU-LOS) and ICU mortality—between COVID-19 and non-COVID-19 critically ill ARDS patients. This was a second analysis of prospectively collected data in four European academic centers pertaining to 101 adult critically ill ARDS patients with and without COVID-19 disease. Depth of sedation (DOS) and delirium were monitored through processed electroencephalogram (EEG) and the Confusion Assessment Method for ICU (CAM-ICU). Our main exposure was excessive sedation and how it relates to the presence of delirium, ICU-LOS and ICU mortality. The criterion for excessive sedation was met in 73 (72.3%) patients; of these, 15 (82.2%) and 58 (69.1%) were in non-COVID-19 and COVID-19 ARDS groups, respectively. The criteria of delirium were met in 44 patients (60.3%). Moreover, excessive sedation was present in 38 (86.4%) patients with delirium (*p* < 0.001). ICU death was ascertained in 41 out of 101 (41.0%) patients; of these, 37 (90.2%) had excessive sedation (*p* < 0.001). The distribution of ICU-LOS among excessive-sedated and non-sedated patients was 22 (16–27) vs. 14 (10.5–19.5) days (*p* < 0.001), respectively. In a multivariable framework, excessive sedation was independently associated with the development of delirium (*p* = 0.001), increased ICU mortality (*p* = 0.009) and longer ICU-LOS (*p* = 0.000), but only in COVID-19 ARDS patients. Independent of age and gender, excessive sedation might represent a risk factor for delirium in COVID-19 ARDS patients. Similarly, excessive sedation shows to be an independent predictor of ICU-LOS and ICU mortality. The use of continuous EEG-based depth of sedation (DOS) monitoring and delirium assessment in critically ill COVID-19 patients is warranted.

## 1. Background

There are various indications for sedation in the intensive care unit (ICU), including patient adaptation to mechanical ventilation, pain/agitation/anxiety management and implementation of invasive procedures. However, if sedation is prolonged or excessive, it can lead to relevant complications, including delirium [1,2,3,4]. Individual response to sedation itself may vary greatly, increasing the risk of oversedation. Although there is a consensus regarding the indication for the use of light instead of deep sedation, quantification of these differences is not always feasible [5]. Recent guidelines recommend that patients should be awake, without pain, anxiety or delirium, provided the clinical condition allows for a “light sedation” approach. This facilitates patients to take an active role in their care and treatment, contributing to a better recovery [6]. However, certain conditions may require deep sedation, such as severe acute respiratory distress syndrome (ARDS), and therefore it becomes necessary to identify the correct balance between the required DOS through precise titration of drug dosages while avoiding the possible correlated complications [6,7,8,9,10,11,12,13,14]. Measurement of DOS in the awake or arousable patient is possible through use of clinical assessment scales (Richmont Agitation Sedation Scale (RASS)), but when sedation causes loss of consciousness, further assessment of DOS is no longer possible [15]. Lower doses of sedatives may be advantageous by avoiding complications due to an excess in sedation [5,16]. During the COVID-19 pandemic, many critically ill ARDS patients required deep sedation throughout their ICU stay. Without a means of measuring the exact DOS, this category of patients may be exposed to further risks, including delirium. Despite intraoperative neurophysiological monitoring being part of standard practice today, clear indication for this type of monitoring in sedated critically ill patients is still lacking. In this regard, this retrospective analysis of prospectively collected data sought to compare the prognostic value of excessive sedation in terms of delirium, ICU-LOS and ICU-mortality and analyze whether these effects varied according to patient’s etiology (COVID-19 vs. non-COVID-19 ARDS).

## 2. Methods

This is a retrospective analysis of data regarding two cohorts, COVID-19 and non-COVID-19 critically ill adult ARDS patients, requiring intubation and sedation for mechanical ventilation who were admitted to the intensive care units of four teaching hospitals, Spedali Civili University Hospital of Brescia, Italy, Addenbrooke’s University Hospital of Cambridge, UK, Hospital Clínic, University of Valencia, Spain, and the Policlinico of San Martino, University of Genoa, Italy, from 1 July 2018 to 19 May 2020.

Due to the observational nature of this study, with data collected retrospectively, in Brescia the study received approval by the “Brescia ethics committee” with number NP-3576 on the 28th of May 2020, when the study had already commenced, and was conducted in accordance with the Declaration of Helsinki. Permission had been granted to retrospectively review the charts of all critically ill patients monitored for the DOS, which represents the standard of care for our center, and for all the patients pertaining to the same category admitted from the date of approval onward, which included the cohort of COVID-19 patients. Consisting of a retrospective dataset analysis of standard-of-care monitoring and sedation, informed consent was waived, and obtained once and if the patient regained mental capacity, since Italian legislation lacks a clear definition of what is considered a legal representative of temporarily incapacitated adult patients. In the Cambridge Addenbrooke’s Hospital, the CUH R&D approved the study on the 14th of July 2020 (Reference: A095650). The Hospital Clínic Universitari de Valencia approved the study on the 9th of July 2020 (Reference: F-CE-GEva-1P V1), and the Genoa University and Liguria Ethics Committee approved this study with protocol number 163/2020. The Strengthening the Reporting of Observational Studies in Epidemiology (STROBE) checklist for data reporting was followed for this study [17].

Inclusion criteria consisted of the following: age >18 years, necessity of deep sedation (defined clinically as a Richmond Agitation Sedation Scale score (RASS) of −4 or below), mechanical ventilation lasting longer than 12 h and patients affected by ARDS (Berlin definition). Patients were excluded if placement of the EEG sensors on the patient’s forehead was not possible due to injury of the scalp and if extensive brain damage itself caused EEG patterns capable of confounding the interpretation of the sedation depth.

## 3. Patient Management

All patients were intubated and mechanically ventilated during the entire monitoring period and arterial blood gas samples were obtained every 6 h in order to maintain a target range of arterial partial pressure of carbon dioxide between 35 and 45 mmHg, when possible. Since all patients had ARDS, due to the different clinical pathophysiological patterns of this disease, various types of ventilation settings were adopted based on the necessity of obtaining and maintaining lung protection strategy. Guidelines for the ventilation of ARDS patients were implemented when appropriate and are described elsewhere [18]. Intravenous propofol or midazolam were used as the main sedatives, followed by dexmedetomidine and ketamine, and for analgesia fentanyl and remifentanil were the main drugs administered. Regarding induced muscle paralysis, the most used neuromuscular blocking agents were rocuronium or cisatracurium, through continuous infusion. All therapeutic strategies adopted during the monitoring periods were aimed at maintaining a stable arterial blood pressure and heart rate and were not modified by this study (Table 1).

## 4. Hemodynamic Monitoring and Neuromonitoring

Systemic hemodynamic monitoring consisted of invasive arterial blood pressure from the radial artery, continuous electrocardiography and pulse oximetry (Edwards Life sciences; Irvine, CA, USA). When necessary, catecholamines (epinephrine and/or norepinephrine) were added in order to reach and maintain adequate end-organ perfusion.

Neuromonitoring consisted of continuous raw and processed EEG (Patient State Index (PSI), Digital Subtraction Array (DSA) and Burst Suppression Ratio (SR)), through use of a four-channel sensor applied to the forehead (Next Generation SedLine^®^, Masimo Corporation, 52 Discovery, Irvine, CA, USA). The minimum continuous cerebral functional monitoring time was 12 h. This monitoring time was based on the fact that all of our critically ill COVID-19 patients who were intubated for respiratory failure received sedation lasting for more than 12 h.

General anesthesia, as defined by the ASA, occurs when a patient is not arousable; therefore, for the purpose of this study, a PSI of 70 to 51 was considered deep sedation and a value of 50 or below general anesthesia [19]^.^ Not having a reference to what may be considered as “excessive sedation” in this category of patients, and to guarantee that only episodes of very deep sedation maintained for a long monitoring period were captured, an excess was considered if the patient had either a PSI <30 or an SR >2 for more than 10% of the total sedation time (TST). Once the patient was weaned from sedation and reached an RASS of -3 or above, delirium was evaluated through use of the Confusion Assessment Method for the ICU (CAM-ICU), which was applied to all patients every 6 h during their ICU stay (Table 1 and Table 2) [20]. The Confusion Assessment Method for the Intensive Care Unit (CAM-ICU) is a structured instrument used to identify delirium in critically ill patients. It consists of four main components: inattention, disorganized thinking, altered level of consciousness and autonomic instability. These components are then used to generate a score of 0–4, with 4 being the most severe. The CAM-ICU showed to have higher accuracy that other scores to evaluate delirium in ICU patients [20].

## 5. Endpoints

The main aim of this study was to determine the association between excessive sedation with the incidence of delirium, in both COVID-19 and non-COVID-19 ARDS patients.

Secondary outcomes were the association between excessive sedation with ICU-LOS and ICU mortality.

## 6. Statistical Analysis

Baseline patient characteristics are reported as means and standard deviations (SDs) for continuous symmetric variables, as medians (interquartile range) for continuous skewed variables and as frequencies (percentages) for categorical variables. Continuous variables were compared among COVID-19 status with either t-tests or rank sum tests for independent samples as appropriate. Discrete variables were compared using the chi-square test.

Delirium as outcome: The effect of excessive sedation on the likelihood of developing delirium was evaluated with logistic regression analysis. Due to small sample size (*n* = 73), the model only included age (years), gender, COVID-19 status and ICU-LOS as adjusting covariates. Age and ICU-LOS were modeled linearly—after linearity assumption tested with a multivariable fractional polynomial. Regression estimates were expressed as odds ratios (OR) with 95% confidence intervals (CIs). Based on prior knowledge regarding the putative role of COVID-19 on delirium, we also tested the interaction between excessive sedation and COVID-19.

Mortality in ICU: For this outcome, the final random effect logistic regression model included (all as main effects) age (years), gender, excessive sedation, COVID-19, ICU-LOS and the variable hospital center (as a random intercept). The interaction between excessive sedation and COVID-19 was also tested by design.

LOS in ICU: Independent predictors for ICU-LOS were determined by linear mixed effect analysis. For this outcome, the final model included (all as main effects) age (years), gender, excessive sedation, COVID-19 and the variable hospital center (as a random intercept). The interaction between excessive sedation and COVID-19 was also tested by design.

A two-sided *p*-value <0.05 was considered statistically significant for all analyses. All analyses were performed with Stata 16.1 (Stata Statistical Software, College Station, TX, USA).

## 7. Results

**Figure 1:**
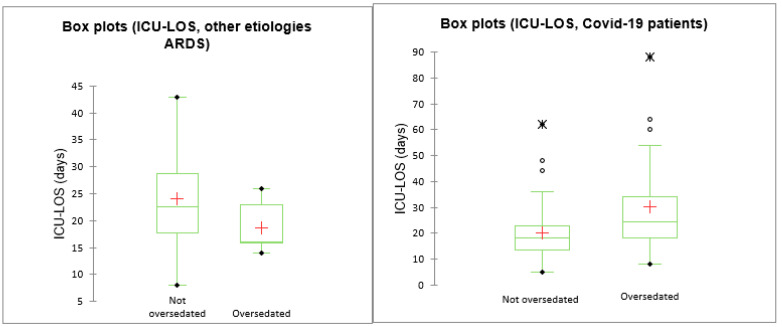
Box plot ICU-LOS and oversedation in COVID-19 patients and other ARDS etiologies.

All enrolled patients (*n* = 101) were diagnosed with ARDS (see Table 2). Of these, 17 (16.8%) and 84 (83.2%) were non-COVID-19 and COVID-19 patients, respectively. The criterion for excessive sedation was met in 73 out of 101 (72.3%) patients, with 15 (82.2%) and 58 (69.1%) belonging to non-COVID-19 and COVID-19 ARDS groups, respectively. Data on delirium were ascertained in 73 out of 101 patients. From this subset, 44 (60.3%) met the criteria for delirium. Moreover, excessive sedation was present in 38 (86.4%) of patients with delirium (*p* < 0.001). ICU death was ascertained in 41 out of 101 (41.0%) of patients; of these, 37 (90.2%) had excessive sedation (*p* < 0.001). The median (IQR) length of stay in the ICU was 20 (14–25) days. The distribution of ICU-LOS among excessive-sedated and non-sedated patients was 22 (16–27) vs. 14 (10.5–19.5) days (*p* < 0.001), respectively. The mean (SD) monitoring time for the COVID-19 and the non-COVID-19 patients was 43 ± 30 and 50 ± 25.6 h, respectively. As shown in Table 2, excessively sedated patients were older (67 ± 10 vs. 58 ± 15, *p* = 0.001) and had delirium more frequently (80.0% vs. 42.1%, *p* = 0.001); there were no differences in COVID-19 diagnosis among excessive-sedated patients (79.5% vs. 92.8%, *p* = 0.107, Table 2). However, when excessive sedation was stratified by COVID-19 and delirium, it was more prevalent in the delirium subgroup (88.6% vs. 26.9%), but only when COVID-19 = 1, and the opposite when COVID-19 = 0 (77.8% vs. 100.0%). In addition, COVID-19 patients received higher doses of neuromuscular blocking agents than the non-COVID-19 population. On unadjusted analysis, ICU delirium was significantly related to excessive sedation and older age, but it did not relate significantly to COVID-19. Concerning outcome, patients with delirium, when compared to patients without delirium, had longer MV duration (*p* = 0.001), ICU-LOS (*p* = 0.000) and H-LOS (*p* = 0.007) (Figure 1). There were no differences in term of SAPS II score between the two groups (*p* = 0.112).

On multivariable logistic regression analysis, excessive sedation—as a main term—was independently related to delirium (OR = 5.97, 95% CI = 1.76–20.24; *p* = 0.004). On further analysis, we found that this effect varied among COVID-19 status (*p*-value for the interaction: 0.012), with a positive and significant association with delirium only in ARDS COVID-19 patients (OR = 10.59, 95% CI (2.48–45.21); *p* = 0.001), but not in those with different etiologies (OR = 0.63, 95% CI (0.02–21.04); *p* = 0.794). For the ICU-LOS outcome, excessive sedation—as a main term—was associated with ICU-LOS (β-coef. = 10.24, 95% CI = 3.71–16.76; *p* = 0.002). The interaction with COVID-19 status (*p*-value for the interaction: 0.009) revealed that excessive sedation was a significant positive predictor of ICU-LOS only in ARDS patients with COVID-19 (β-coef. = 13.03, 95% CI (6.36–19.70); *p* <0.001), but not in non-COVID-19 patients (β-coef. = −15.39, 95% CI (-35.55–4.78); *p* = 0.135). For the ICU mortality outcome, excessive sedation—as a main term—was associated with higher mortality (OR = 4.59, 95% CI = 1.29–16.26; *p* = 0.018). However, looking at the effect among COVID-19 status (*p*-value for the interaction: 0.143), excessive sedation was a significant predictor only in ARDS patients with COVID-19 (OR = 6.78, 95% CI (1.62–28.28); *p* = 0.009), but not in non-COVID-19 patients (OR = 0.50, 95% CI (0.023–11.24); *p* = 0.665) (Table 1, Table 2, Table 3 and Table 4).

## 8. Discussion

In the present study, we found that excessive sedation is a risk factor for delirium development in critically ill ARDS patients, independent of age, gender or ICU-LOS. Delirium was strongly associated to longer MV, H-LOS and ICU-LOS. Moreover, oversedation predicted ICU-LOS and ICU mortality, but not H-LOS, after adjusting for age, gender, excessive sedation and COVID-19 diagnosis (Figure 1).

A great effort has been made in the last decade towards sedation monitoring. Monitoring of the sedation depth during anesthesia has been made possible through use of processed EEG, which initially found its place as a monitoring system to unmask undersedation and awareness. Recently, continuous EEG monitoring has been introduced to monitor depth of sedation also in the ICU environment, yet it still lacks consensus among ICU clinicians [21,22].

Although the literature suggests lighter sedation targets in critically ill patients, certain conditions which require the patient to be deeply sedated still exist, as for ARDS patients. In general, severe ARDS patients frequently require neuromuscular blocking agents for adaptation to mechanical ventilation. It would be unethical to paralyze patients without sedation, therefore all patients who require NMBA will also require deep sedation. The most commonly used sedatives for long-term sedation in ICUs are propofol and midazolam, and during the COVID-19 crisis many countries experienced a shortage of these sedatives [23]. The use of benzodiazepines has been shown to be an independent risk factor for delirium in critically ill patients, as is heavy sedation in general [2]. It becomes therefore paramount to limit the use of sedatives to the minimum dose necessary in order to obtain the desired effect.

There is a substantial amount of literature which associates a rather high complication rate in critically ill mechanically ventilated patients undergoing deep and/or prolonged sedation [12,13,14,15,16]. In our center in Brescia Lombardy, monitoring of the depth of sedation and delirium of critically ill patients was already in progress when the coronavirus crisis arrived, and later continued as they were substituted by mechanically ventilated COVID-19 patients requiring deep sedation and neuromuscular blocking agents. This study used cerebral functional monitoring to demonstrate an elevated incidence of excessive sedation and delirium in both critically ill COVID-19 and non-COVID-19 ARDS patients. Among the findings, excessive sedation was still significantly associated with a higher probability of delirium, despite adjusting for age, and were both more frequent in ARDS patients compared to those without ARDS. The COVID-19 patients had a higher incidence of delirium; however, due to the scarce number of these patients, we were not able to attribute COVID-19 as being an independent risk factor. COVID-19 critically ill patients had lower median and mean SAPS II scores than the non-COVID-19 cohort. Despite this, there was a statistically significant increase in LOS-ICU, LOS-HOS and MV duration in the COVID-19 cohort compared to the non-COVID-19 cohort, both with and without ARDS.

## 9. Conclusions

This study shows that in addition to age, excessive sedation represents an important risk factor for delirium in both COVID-19 and non-COVID-19 critically ill ARDS patients undergoing deep sedation, and that this may lead to an increased ICU-LOS, H-LOS and days of MV. Both excessive sedation and delirium were more common when patients of both cohorts had ARDS. Since critically ill COVID-19 patients frequently have ARDS, this study suggests the use of continuous EEG-based monitoring systems for the quantification of sedation depth along with frequent delirium assessment in this category of patients. In order to confirm whether this monitoring-based strategy leads to improved outcome, larger, randomized and interventional trials are warranted.

## 10. Study Limitations

The main study limitations of this study consist of a small study population and that the two cohorts of patients pertained to two different study recruitment periods, before and after the start of the SARS-CoV2 pandemic. We also acknowledge the fact that the thresholds used to define excessive sedation were based on the monitor’s manufacturers indications and not on validation studies.

## Figures and Tables

**Table 1:** Demographic and clinical characteristics of the study population.

Variables	No COVID-19	COVID-19	Total	*p*-Value
Number of Patients (%)	17 (16.83)	84 (83.17)	101 (100)	*********
**Study Centers**				
Spedali Civili Hospital—University of Brescia, *n* (%)	17 (100.00)	11 (13.10)	28 (27.72)	*********
Addenbrooke’s Hospital—University of Cambridge, *n* (%)	0 (0.00)	10 (11.90)	10 (9.90)	*********
San Martino Hospital—University of Genoa, *n* (%)	0 (0.00)	21 (25.00)	21 (20.79)	*********
Hospital Clínic Universitari—University of Valencia, *n* (%)	0 (0.00)	42 (50.00)	42 (41.58)	*********
**Baseline Demographics**				
Patient age, years, mean (SD)	67 (15)	62 (13)	63 (13)	0.163
Gender male (M), *n* (%)	10 (58.82)	57 (67.86)	67 (66.34)	0.472
Indicator for delirium (Yes), *n* (%)	9 (75.00)	35 (57.38)	44 (60.27)	0.254
Simplified Acute Physiology Score II, mean (SD)	45 (14)	39 (10)	40 (11)	0.078
Simplified Acute Physiology Score II, median (IQR)	47 (32; 58)	38 (32; 45)	38 (32; 47)	0.112
**Sedative Medications**				
Ketamine (yes), *n* (%)	1 (5.88)	21 (28.38)	22 (24.18)	0.051
Propofol mg/h, mean (SD)	42 (69)	124 (55)	108 (65)	0.000
Midazolam mg/h, mean (SD)	3.29 (2.39)	3.39 (4.88)	3.37 (4.51)	0.936
Fentanyl mcg/kg/h, mean (SD)	0.82 (0.58)	0.27 (0.32)	0.37 (0.44)	0.000
Morphine mg/h, mean (SD)	0.06 (0.24)	0.00 (0.00)	0.01 (0.10)	0.036
Remifentanil mcg/kg/min, mean (SD)	0.00 (0.00)	0.12 (0.14)	0.10 (0.14)	0.001
Dexmedetomidine mcg/kg/h, mean (SD)	0.02 (0.08)	0.22 (0.40)	0.18 (0.37)	0.047
**Neuromuscular Blocking Agents**				
None, *n* (%)	2 (11.76)	22 (29.73)	24 (26.37)	*********
Rocuronium, *n* (%)	0 (0.00)	48 (64.86)	48 (52.75)	*********
Cisatracurium, *n* (%)	15 (88.24)	4 (5.41)	19 (20.88)	*********
Use neuromuscular blocking agents, *n* (%)	15 (88.24)	52 (70.27)	67 (73.63)	0.130
Dose of neuromuscular blocking agents, mean (SD)	7.59 (3.95)	32.03 (24.20)	27.46 (23.86)	0.000
**Richmont Agitation Sedation Scale**				
Richmont Agitation Sedation Scale, mean (SD)	−4.7 (0.8)	−4.6 (0.8)	−4.6 (0.8)	0.657
**Oversedation Parameters**				
Oversedation indicator—when either criterion, *n* (%)	15 (88.24)	58 (69.05)	73 (72.28)	0.107
Oversedation indicator—when both criteria, *n* (%)	12 (70.59)	40 (47.62)	52 (51.49)	0.084
**Length of Stay**				
Length of stay in ICU, days, median (IQR)	16 (14; 23)	21 (14; 26)	20 (14; 25)	0.212
Length of stay in hospital, days, median (IQR)	24 (18; 46)	28 (22; 42)	28 (21; 44)	0.654
**Main Clinical Outcomes**				
Duration of mechanical ventilation, days, median (IQR)	13 (7; 20)	18 (9; 25)	15 (9; 25)	0.095
Indicator for death in ICU, *n* (%)	7 (41.18)	34 (40.48)	41 (40.59)	0.957

Legend: ICU, intensive care unit; SD, standard deviation; IQR, interquartile range.

**Table 2:** The regression estimates table for predictors of delirium.

Covariates	Main Terms Model			Interaction Model		
	OR	95% CI	*p*-Value	OR	95% CI	*p*-Value
**COVID-19 status**	0.588	0.135–2.565	0.479			
**Patient age, per 5 years increase**	1.195	0.947–1.507	0.133	1.151	0.908–1.459	0.244
**Gender**	3.081	0.908–10.452	0.071	3.329	0.945–11.723	0.061
**ICU length of stay, per 5 days**	1.261	0.965–1.648	0.090	1.164	0.897–1.511	0.253
**^1^ Indicator for excessive sedation**	5.965	1.758–20.240	0.004			
**^2^ Interaction term**						0.012
**Excessive sedation**				0.627	0.019–21.043	0.794
**ARDS_COVID-19**				0.078	0.003–2.438	0.146
Excessive sedation*COVID-19 status				16.903	0.327–872.697	0.160
***n***	73			73		

Legend: ARDS, acute respiratory distress syndrome; ICU, intensive care unit; CI, confidence interval.

**Table 3:** The regression estimates table for ICU-LOS.

Covariates	Main Terms Model			Interaction Model		
	β-Coef.	95% CI	*p*-Value	β-Coef.	95% CI	*p*-Value
**Patient age, years**	−0.110	−0.334–0.113	0.333			
**Gender male**	0.143	−5.681–5.966	0.962	0.386	−5.270–6.041	0.894
**COVID-19 status**	6.252	−1.232–13.735	0.102			
**^1^ Indicator for excessive sedation**	10.236	3.710–16.762	0.002			
**Patient age, per 5 years increase**				−0.693	−1.782–0.396	0.212
**^2^ Interaction term**						
**COVID-19 status**				−18.449	−38.281–1.384	0.068
**Excessive sedation**				−15.388	−35.552–4.777	0.135
Excessive sedation*COVID-19 status				28.422	7.187–49.656	0.009
**Constant**	16.672	0.541–32.803	0.043	41.022	17.019–65.025	0.001
***n***	101			101		

Legend: ICU-LOS, intensive care unit length of stay; CI, confidence interval; *n*, number.

**Table 4:** The regression estimates table for mortality.

Covariates	Main Effect			Interaction Term		
	OR	95% CI	*p*-Value	OR	95% CI	*p*-Value
**COVID-19 status**	1.655	0.477–5.743	0.428			
**Patient age, per 5 years increase**	1.431	1.127–1.817	0.003	1.434	1.123–1.832	0.004
**Gender**	1.588	0.606–4.161	0.346	1.650	0.625–4.356	0.312
**^1^ Indicator for excessive sedation**	4.586	1.294–16.257	0.018			
**ICU length of stay, per 5 days**	1.018	0.872–1.188	0.822	0.994	0.848–1.165	0.939
**^2^ Interaction term**						
**COVID-19 status**				0.174	0.007–4.145	0.280
**Excessive sedation**				0.504	0.023–11.235	0.665
Excessive sedation*COVID-199 status				13.454	0.417–434.494	0.143
***n***	101			101		

Legend: OR, odds ratio; CI, confidence interval.

## Data Availability

Not applicable.
